# Targeting MIAT reduces apoptosis of cardiomyocytes after ischemia/reperfusion injury

**DOI:** 10.1080/21655979.2019.1605812

**Published:** 2019-04-30

**Authors:** Longying Chen, Dianlong Zhang, Li Yu, He Dong

**Affiliations:** a Department of Internal medicine intensive care, the central hospital of Linyi, Yishui, Shandong, China; b Department of Anesthesia, the affiliated hospital of Qingdao University, Qingdao Shandong, China

**Keywords:** Hypoxia/reoxygenation, ischaemia-reperfusion, apoptosis, lncRNA myocardial infarction-associated transcript, nuclear factor kappa B, p53 upregulated modulator of apoptosis

## Abstract

This study aims to investigate the role of targeting lncRNA myocardial infarction-associated transcript (MIAT) in protection against hypoxia/reoxygenation (H/R) injury in H9c2 cells *in vitro* and myocardial ischemia/reperfusion (I/R) injury *in vivo* by regulating expression of NF-kB and p53 upregulated modulator of apoptosis (PUMA). H9C2 cells were infected with lentivirus expressing the short-hairpin RNA direct against human MIAT gene (Lv-MIAT shRNA) or lentivirus expressing scrambled control (Lv-NC shRNA) or PUMA siRNA or p65 siRNA or their control siRNA respectively. Then the H9c2 cells were infected with Lv-shRNA to 2 hours of hypoxia (H) and 24 hour of reoxygenation (R). 100 ul of Lv-MIAT shRNA (1 × 10^8^ PFU) or Lv-NC shRNA was transfected into mouse hearts, then the hearts were subjected to I/R (1h/72 h). We discovered targeting MIAT remarkably enhanced H9c2 cell viability, decreased H/R-induced cell apoptosis and LDH leakage and significantly decreased I/R-induced myocardial infarct size, reduced myocardial apoptosis and enhanced the heart function. Targeting MIAT downregulated p65 nuclear translocation, NF-κB activity and anti-apoptotic protein cleaved-caspase-3, Bax, and upregulated anti-apoptotic protein Bcl-2 induced by H/R or I/R. Our study suggests that targeting MIAT may protect against H9c2 cardiomyoblasts H/R injury or myocardial I/R injury via inhibition of cell apoptosis, mediated by NF-κB and PUMA signal pathway.

## Introduction

Ischemia induced by the interruption of heart blood flow evokes significant cardiac myocytes damages. Paradoxically, the subsequent reperfusion also activates various injury responses and further tissue lesions what is known as ischemia and reperfusion (I/R) injuries [1]. Advances in the understanding of pathophysiological mechanisms of I/R injury has identified a number of possible therapeutic targets and cardioprotectors [2]. A more complete understanding of cardiomyocyte apoptosis holds great therapeutic potential to the medical community due to the phenomena’s prominent role in a variety of cardiac diseases, including acute myocardial infarction (AMI) rat, heart failure, and cardiac allograft [3]. In the search for a mechanism, long non-coding RNAs (lncRNAs) are of particular interest due to their demonstrated effect on cell proliferation and apoptosis in cardiology.

Long noncoding RNAs (lncRNAs) are RNA transcripts longer than 200 nucleotides which, although not having the function of direct coding proteins, can regulate the expression of genes at transcriptional, post-transcriptional, and translational levels [4,5]. MIAT, also termed as Gomafu in human or Rncr2 in mouse [6–8], is a promising functional factor among all disease-associated lncRNAs, and exhibits deregulation in multiple diseases, including up-regulation in ischemic stroke, myocardial infarction, neuroendocrine prostate cancer, non-small-cell lung cancer, diabetic cardiomyopathy, cataract, chronic chagas disease cardiomyopathy, chronic lymphocytic leukemia and down-regulation in schizophrenia, diabetic nephropathy, bone disease [9]. It has recently found that MIAT is upregulated in hearts of a mouse model of AMI, and knocking it down improves cardiac function by inhibition of activation of NF-κB signaling pathway [10]. Furthermore, knockdown of *MIAT* improved cardiac functions, decreased cardiomyocytes apoptosis and attenuated inflammatory cell infiltration in vivo [11]. However, the underlying mechanisms remains unclear.

PUMA (p53 upregulated modulator of apoptosis) is a BH3-only Bcl-2 family member which functions as a critical initiator of apoptosis in cancer cells [12]. *PUMA* was also markedly induced in cardiomyocytes following cardiac I/R [13]. Furthermore, after I/R, cardiac function was significantly better preserved in PUMA(-/-) mice than in their wild-type littermates [14]. Cardiac I/R could activate NF-κB, and pharmacological inhibition of NF-κB significantly ameliorated infarct formation in WT mice, implicating acute activation of NF-κB in the pathogenesis of reperfusion injury [15]. Wang et al. has reported that PUMA is directly activated by NF-κB in response to TNF-α treatment in a p53-independent manner [16]. Zhang et al. has reported that NF-κB/PUMA signaling pathway was activated during acute cerebral I/R injury, and pharmacological inhibition of apoptosis through suppression of NF-κB/PUMA signaling pathway had neuro-protective effects [17].

The present study was designed to investigate the effect and mechanisms of targeting *MIAT* on H9c2 cardiomyocytes following H/R in vitro and heart I/R injury in vivo. These results indicate that the cardiomyo-protective effects of targeting *MIAT* following cardiac H/R and I/R injury are possibly related to the inhibition of apoptosis through suppression of NF-κB and PUMA signaling pathway.

## Materials and methods

### Ethics statement

The study was conducted in accordance with the ethical standards and the Declaration of Helsinki and according to the national and international guidelines and was approved by the central hospital of Linyi, Yishui, Shandong, China.

### Construction of lentiviral vector

The short-hairpin RNA direct against human MIAT gene (MIAT shRNA) was constructed into the lentivirus expression vector using a lentivirus expressing system (Shanghai, China) as the manufacture’s instruction. Sequences of MIAT shRNA: Sence,5′-GATCCCCGGACA GAGAATGCAAATAATTCAAGAG ATTATTTGCATTC TCTGTCCTTTTTA-3′, (reverse); non-sense MIAT sequence control (NC shRNA): Sense, CACCGTTCTCCGAACGTGTCACGTTTCAGAGAACGTGACACGTTCGGAGAATTTTTTG,antisence,GATCCAAAAAATTCTCCGAACGTGTCACGTTCTCTTGAAACGTGACACGTTCGAGAA C. The lentiviral control vector contains a non-sense MIAT sequence (NC shRNA). Recombinant lentiviruses were produced by transfecting 293T cells with the lentiviral expression plasmid CN362 and the packaging plasmids that are psPAX2 of gag/pol and pMD2.G of VSV-G using Frans-EZ (SunBio, Shanghai) reagent. 293T cells (6 × 10^5^) were cultured in a 10-cm tissue culture plate with opti-MEM (GIBCO, USA). Transfection was performed when the cell density reached 30%–40% confluency. Solution A was prepared by adding 0.5 ml (0.5 mg/ml) CN362 plasmid, 1 ml (0.2 mg/ml) PMD2.G and 0.5 ml (0.2 mg/ml) psPAX2 plasmids (diluted by opti-MEM medium), and then Opti-MEM medium to 18 ml in a 50-ml tube. Solution B was prepared by adding 0.5 ml Frans-EZ in another 50-ml tube and then Opti-MEM medium to 18 ml. Transfection solution was prepared by slowly adding solution B to solution A. The mixture was agitated and then left in the hood at room temperature for 20 min. Three ml of the prepared transfection mixture was added to a plate of 293T cells and the cells were cultured routinely. After 6 hours of culture, the culture medium was exchanged with Dulbecco’s modified Eagle’s medium (DMEM) (GIBCO, USA). Infectious lentiviruses were harvested at 48 hours post-transfection and then centrifuged at 4000 g, 4°C for 10 min and incubated with 5 u/ml DNaseI (Promega, USA) and 10 mM MgCl_2_ (Sigma, UK) for 30 minutes. The vector was then aliquoted and stored at −80°C. The lentiviral titre was determined by serial dilution and transduction of 293T cells. We counted the numbers of clusters of GFP-positive 293T cells 48 h after transduction. Prior to use, all the lentiviral vectors (Lv- MIAT shRNA or Lv- NC shRNA) were titre matched to 1 × 10^8^ transducing units/ml.

### In vitro *Hypoxia/Reoxygenation(H/R) model*

H9C2 cells were plated in a 6 well plate (80,000 cells/well) and cultured in DMEM medium supplemented with 10% fetal bovine serum and 100 U/ml penicillin and streptomycin overnight. DMEM culture medium was replaced by deoxygenized PBS for 2h followed by a normal culture environment with 5% CO2 and 28% O^2^ at 37°C for 24 h. The cells that were not subjected to H/R served as control (normoxia).

### In vitro *shRNA infection and siRNA transfection*

For cell infection, one day before infection, H9C2 cells were split to 6 well-culture dishes at a density of 2.0 × 10^5^ cells per well. The confluency of H9C2 cells was approximately 70% at the time of infection. Then the H9C2 cells were infected into Lv- MIAT shRNA or Lv- NC shRNA (3.6 × 10^5^ IFU) for 48 h.

To study the effect of MIAT on ischemia reperfusion-induced cell injury, H9C2 cells were infected into Lv- MIAT shRNA or Lv- NC shRNA (3.6 × 10^5^ IFU) for 24 h, then the shRNA-transfected H9C2 cells were subjected to H/R as the methods of *In vitro* ischemia reperfusion model above. The cells were harvested at 24 h for isolation of cellular protein.

To study the effect of P65 or PUMA on ischemia reperfusion-induced cell injury, H9C2 cells were transfected with PUMA siRNA or p65 siRNA or their control siRNA (Dharmacon, Chicago, IL, USA) for 24 h following the manufacturer’s instructions. Then the siRNA-transfected H9C2 cells were subjected to H/R as the methods of *In vitro* ischemia reperfusion model above. The cells were harvested at 24 h for isolation of cellular protein.

### Analysis of LDH leakage and cell viability

Cell injury was evaluated by lactate dehydrogenase (LDH) leakage. The released LDH in the collected medium was determined by using a commercial LDH kit (Roche, Mannheim, Germany) according to the manufacturer’s instructions. To demonstrate bioactivity of MIAT, H9C2 cells at a density of 1 × 10^4^ cells per well in 96-well plates were grown in 10% fetal bovine serum and 100 U/ml penicillin and streptomycin, and infected with Lv- MIAT shRNA or Lv- NC shRNA (3.6 × 10^5^ IFU) for 24 h. The shRNA-transfected H9C2 cells were subjected to hypoxia for 2 h followed by reoxygenation (H/R) for 24 h. Then the H9C2 cells were incubated with 3-(4, 5-dimethylthiazol-2-yl) −2, 5-diphenyl-tetrazolium-bromide (MTT) (0.5 mg/ml) at 37oC for 3 h. Finally, dimethyl sulfoxide (DMSO) solution (100 mM) was added to dissolve formazan crystals. The absorbance was detected at 570 nm wavelength using a microplate reader (Molecular Devices).

### In vitro *Caspase-3/7 activity assay*

Caspase 3/7 activities were measured using Caspase-Glo 3/7 Assay (Promega, Annandale, NSW, AUS) according to manufacturer’s instructions. Briefly, H9C2 cells were plated at density of 8 × 10^3^ cells/well in a 96 well plate. Cells were incubated for overnight and infected with Lv- MIAT shRNA or Lv- NC shRNA (3.6 × 10^5^ IFU) for 24 h or transfected with PUMA siRNA or p65 siRNA or their control siRNA for 24 h. Then the shRNA/siRNA-transfected H9C2 cells were subjected to hypoxia for 2 h followed by reoxygenation (H/R) for 24 h. Later, the Caspase-Glo 3/7 reagent was added to the cells (1:1). Incubation time was optimized at 30 min and caspase 3/7 luminescence as relative light units (RLU) was measured using the Synergy 2 Multi-Mode microplate reader. The average background reading of control wells were subtracted from all values and final values were normalized to protein content.

### In vivo infection of Lv-shRNA into mouse hearts

Mice were intubated and anaesthetized with mechanical ventilation using 5% isoflurane. Anaesthesia was maintained by inhalation of 1.5–2% isoflurane in 100% oxygen. The adequacy of anaesthesia was monitored by measuring heart rate and the response to tail stimulation. Body temperature was maintained at 37°C by surface water heating. An incision was made in the middle of the neck and the right common carotid artery was carefully exposed. The common carotid artery was isolated by temporary ligation of the proximal common carotid artery and proximal internal carotid artery. A micro-catheter was introduced into the isolated common carotid artery and positioned into the aortic root. 100 ul of Lv-MIAT shRNA (1 × 10^8^ PFU) or Lv-NC shRNA was injected through the micro-catheter. The micro-catheter was gently removed and the common carotid artery was tightened before the skin was closed. After the left ventricle (LV) was exposed, a 30-gauge needle was advanced from the apex of LV along to the left anterior free wall adjacent to the ligated area. There were four injections with a total volume of 20 µL of Lv- MIAT shRNA or Lv-NC shRNA. Five days after infection, the hearts were harvested. The expression of MIAT and bcl-2 in the heart tissues was examined by qPCR or Western blot.

### In vivo *myocardial I/R model*

Myocardial I/R injury was induced 5 days after transfection of Lv-MIAT shRNA or Lv-NC shRNA. Briefly, the mice were anesthetized by 5.0% isoflurane, intubated and ventilated using a rodent ventilator. Anesthesia was maintained by inhalation of 1.5% to 2% isoflurane driven by 100% oxygen flow. Body temperature was regulated at 37°C by surface water heating. The hearts were exposed and the left anterior descending (LAD) coronary artery was ligated with an 8–0 silk ligature. After completion of 60 min of occlusion, the coronary artery was reperfused by releasing the knot of suture four 72 hours. During reperfusion, cardiac function was measured by echocardiography. After reperfusion for the time indicated, the mice were euthanized by CO2 inhalation and the hearts were harvested. Infarct size was evaluated by triphenyltetrazolium chloride (TTC, Sigma-Aldrich) staining .

### Western blot analysis

In vitro in H9C2 cells, after shRNA or siRNA infection/transfection followed by hypoxia-reoxygenation, cells were lysed in Radio Immuno Precipitation Assay (RIPA) buffer. Whole cell extracts were prepared using lysis buffer containing 20 mM 4-(2-hydroxyethyl)-1-piperazineethanesulfonic acid (HEPES) (pH 7.9), 400 mM NaCl, 10 mM KCl, 1 mM ethylenediaminetetraacetic acid (EDTA) (pH 8.0), 0.5% Nonidet P-40 (NP-40), 1 mM phenylmethylsulfonyl fluoride (PMSF), 1 m*M* dithiothreitol (DTT), 1 mM Na_3_VO_4_, 5 mM NaF, 20% glycerol, and 1% (v/v) mammalian protease inhibitor (Sigma-Aldrich). ***In vivo* in mouse hearts**, tissues were homogenized in lysis buffer containing 50 mM Tris-HCl (pH 8.0), 150 mM NaCl, 0.1% sodium dodecyl sulfate (SDS), 1% Triton X-100, 0.02% sodium azide, and 1% protease inhibitor cocktail (Sigma-Aldrich).

Protein content was determined by the Bradford method. The proteins were stored at −80 °С for western blot analysis. Samples (50 μg) were mixed with Laemmli buffer containing 0.125 M Tris (pH 6.8), 20% glycerol, 10% beta-mercaptoethanol, 4% SDS, and 0.002% bromophenol blue, and heated at 95°C for 5 min. Protein was loaded on a 10% sodium dodecyl sulfate polyacrylamide gel electrophoresis (SDS-PAGE) gel and separated by electrophoresis using a Bio-Rad system with molecular weight standards (Rainbow-GE) at 50 V for 20 min and 90 V for 1 h. Proteins were transferred to a membrane in transfer buffer at 80 V for 1.5 hours. The membrane was blocked with 5% skim milk in Tris-buffered saline with Tween (TBS-T) solution for 30 minutes at room temperature, and incubated with a diluted solution of primary antibody(anti-p65, PUMA, BCL-2, Bax, cleaved-caspase-3)(1:200) (Cell Signaling Technology, Danvers, MA) and mouse anti-β-actin (1:500) (Sigma) antibodies overnight at 4℃. Following washing in TBS-T, the membrane was incubated with secondary antibody solution (anti-rabbit, 1:2000 dilution) for 1 hour at room temperature. The signals were visualized using the ECL detection system (Amersham, Chalfont, UK). The intensity (area x density) of individual bands was measured by densitometry (model GS-700, Imaging Densitometer; Bio-Rad, Hercules, CA), and the background was subtracted from the calculated area.

### Electrophoresis mobility shift assay (EMSA) of NF-κB activation

In vitro, nuclear extracts (NE) were prepared from H9C2 cells after shRNA or siRNA infection/transfection followed by hypoxia-reoxygenation as described previously [18]. Cell pellets were resuspended in hypotonic buffer (10 mM HEPES, pH 7.9, 1.5 mM MgCl_2_, 10 mM KCl, 0.2 mM PMSF, 0.5 mM DTT, 10 µg·mL^−1^ aprotinin) and incubated on ice for 15 min. Cells were then lysed by adding 0.1% Nonidet P-40 and vortexed vigorously for 10 s. Nuclei were pelleted by centrifugation at 12 000 × g for 1 min at 4°C and resuspended in high salt buffer (20 mM HEPES, pH 7.9, 25% glycerol, 400 mM KCl, 1.5 mM MgCl_2_, 0.2 mM EDTA, 0.5 mM DTT, 1 mM NaF, 1 mM sodium orthovanadate).

In vivo, Nuclear fractions were prepared as described previously [19]. All steps were carried out on ice. Tissue samples were homogenized in 0.5 ml of buffer A (10 mmol/L N-2-hydroxyethylpiperazine-N′-2-ethanesulfonic acid [pH 7.9], 1.5 mmol/L MgCl_2_, 10 mmol/L KCl, 1 mmol/L dithiothreitol, and 1 mmol/L phenylmethanesulfonylfluoride [PMSF]), incubated for 10 minutes, and then centrifuged at 850*g* for 10 minutes at 4°C. The pellets were resuspended in 1.5 times cell volume of buffer A with 0.1% Triton X-100, incubated for 10 minutes, and centrifuged as mentioned earlier. The supernatant was removed and saved as the cytoplasmic fraction. The pellet was resuspended in 300 µl of buffer A, centrifuged as mentioned earlier, and resuspended in 1 cell volume of a buffer of 20 mmol/L*N*-2-hydroxyethylpiperazine-*N*′-2-ethanesulfonic acid (pH 7.9), 25% glycerol (volume per volume), 420 mmol/L NaCl, 1.5 mmol/L MgCl_2_, and 0.2 mmol/L EDTA. After incubation for 30 minutes, the nuclear fraction was recovered by centrifugation at 20,000*g* for 15 minutes at 4°C.

Fractions were assayed for protein concentration (BCA Protein Assay Kit, Pierce, Rockford, IL) and stored at −80°C until analysis. The sequence of NF-κB/p65 oligonucleotides used for EMSA was 5′-CAT CGG AAA TTT CCG GAA ATT TCC GGA AAT TTC CGG C-3′/5′-GCC GGA AAT TTC TGG AAA TTT CCG GAA ATT TCC AT G-3′. Protein/nucleic acid complexes were resolved using 5% non-denatured polyacrylamide gel and transferred to Biodyne™ B nylon membrane (Thermo Scientific) in 0 · 5 × Tris–borate–EDTA (TBE) buffer. The biotin-labelled NF-κB/p65 probe was exposed by using a streptavidin–horseradish peroxidase conjugate and chemiluminescence substrate.

### Histopathological analysis

Heart biopsies were taken from the left ventricle. The heart samples were fixed in formaldehyde solution (10% in phosphate buffered saline) at room temperature, dehydrated by graded ethanol and embedded. Heart sections

(thickness 7μm) were deparaffinised with xylene stained with haematoxylin/eosin and examined by light microscopy (Dialux 22 Leitz). All histological studies were executed in a blinded manner. The scoring method was chosen based on previous studies using the following criteria: 0, no damage; 1 (minor), focal swelling and necrosis of the myocytes; 2 (severe), necrosis with evidence of neutrophil infiltration in the myocytes; 3 (major), necrosis with massive neutrophil infiltration.

### Determination of myocardial infarct size by histological staining

At the end of the experiment, 1.5 ml of 2% Evans blue dye was perfused into the LV cavity to stain the non-ischemic region (area not at risk). The left ventricles were cut into five 2 mm transverse slices and incubated in 1% TTC in phosphate buffer (pH 7.4, 37°C) for 20 min. The non-infarcted myocardium was deep red, in contrast to the pale white of the infarcted myocardium. Infarcted and non-infarcted myocardium were digitally measured using an image analysis software (ImageJ, version 1.6, National Institutes of Health). The ratio of infarct size (white)/total area (white plus red) was used to compare the differences among groups.

### Terminal deoxynucleotidyl transferase (tdt)-mediated deoxyuridine triphosphate (dutp)-biotin nick end-labeling (TUNEL) assay for myocardial apoptosis

Hearts were stored in a 10% formalin solution, and paraffin embedded tissue section was mounted on glass slides. Apoptosis was then assessed in the transverse sections of paraffin sections as previously reported. Apoptotic cells were examined under a fluorescence microscope (Nikon Eclipse T*i*) which were clearly identified with a strong nuclear green fluorescence. All cell nuclei were visualized as blue fluorescence following staining with DAPI. The apoptotic index was expressed as the number of apoptotic cells of all cardiomyocytes per field. Apoptotic rate in the peri-infarct regions was calculated using 6 random fields. TUNEL-positive cells (%) is calculated as the ratio of the number of TUNEL-positive cell nuclei divided by the number of total cell nuclei.

### Determination of cardiac function

The left ventricular function prior to the I/R protocol was assessed using an echocardiograph. Fractional shortening (FS) was calculated using the formula: % FS = [left ventricular internal dimensions during diastole (LVPWd)-left ventricular internal dimensions during systole (LVIDs)] x 100/LVIDd. At the end of the reperfusion period, LV fractional shortening (FS), Left ventricular (LV) end-diastolic diameter (LVEDD) and end-systolic diameter (LVESD), and ejection fraction (EF)

### Measurement of serum creatine kinase

After the 2 h reperfusion period, 1 mL of blood samples were obtained from the right carotid artery, and was placed at RT for 0.5 h and centrifuged at 1000 rev min-1 for 10 min and stored at −80°C for use. Supernatants were then obtained. Creatine kinase mb isoenzyme (CK-MB) was measured using CK-MB Assay Kits (Sigma-Aldrich, Shanghai, China) according to the manufacturer’s instruction.

### Reverse transcription quantitative polymerase chain reaction (qRT-PCR) assay

Total RNA was extracted from cells by using Trizol reagent (Invitrogen, Carlsbad, CA, USA), according to the manufacturer’s protocol. The nuclear and cytoplasmic fractions were separated by PARIS Kit (Life Technologies, Foster City, CA, USA), as described by the manufacturer. SYBR Premix Ex Taq and TaqMan gene expression assays (Applied Biosystems, Foster City, CA, USA) were used for the detections of MIAT and Glycolytic glyceraldehyde-3-phosphate dehydrogenase (GAPDH). Primers used for quantitative PCR with reverse transcription were shown below: MIAT,F: AGGTCAGGCAGAGGAAGTCA, R:CTCCCGATACAACAATCACG. GAPDH, 5′-TGCCCAGAACATCATCCCT-3′ and 5′-GGTCCTCAGTGTAGCCCAAG-3′. Relative expression values were normalized and calculated using the relative quantification (2^−△△Ct^) method.

### Statistical analysis

All data are expressed as the mean ± SD. The results were analyzed by unpaired Student’s *t* test or by one-way ANOVA followed by Bonferroni’s post hoc tests when multiple experimental groups were compared. A *p*-value of *p* < 0.05 was considered significant.

## Results

### Transfection of Lv-MIAT shRNA attenuates H/R-induced injury in H9C2 cells

**Figure 1.**
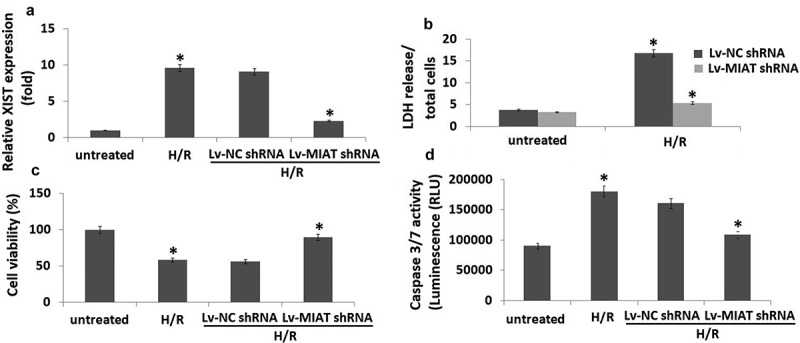
Targeting MIAT mediated cardioprotective effects in H/R-induced H9C2 cardiomyocytes apoptosis. H9C2 cells were infected with Lv- MIAT shRNA or Lv-NC shRNA 24 h, then subjected to H/R (2/24 h). a, MIAT expression was detected in H9C2 cells by qRT-PCR. b, The release of LDH (cell injury marker) in H9C2 cells. c, cell viability was detected by MTT. d, Cell apoptosis was detected by caspase-3/7 activity assay. *P < 0.05 compared with indicated groups.

As shown in Figure 1(a), expression of MIAT was significantly increased in the H9C2 cells subjected to hypoxia for 2 h followed by reoxygenation (H/R) for 24 h compared to the untreated H9C2 cells. And Lv-MIAT shRNA transfection significantly decreased MIAT expression in the H9C2 cells subjected to H/R compared to the Lv-NC shRNA-transfected H9C2 cells subjected to H/R. LDH activity increased 4.6-fold in the H9C2 cells subjected to H/R, but Lv-MIAT shRNA transfection significantly attenuated the LDH activity in the H9C2 cells subjected to H/R (Figure 1(b)). Lv-NC shRNA transfection did not affect LDH activity in H9C2 cells subjected to H/R.

Figure 1(c) showed that H/R markedly decreased cell viability (42%) compared to the untreated normoxic cells. However, H/R did not significantly affect cell viability in the Lv- MIAT shRNA-transfected H9C2 cells subjected to H/R compared to the Lv-NC shRNA-transfected H9C2 cells subjected to H/R. In addition, transfection of Lv-MIAT shRNA attenuated caspase-3/7 activity by 40% in the H9C2 cells subjected to H/R compared to the Lv-NC shRNA-transfected H9C2 cells subjected to H/R (Figure 1(d)).

### Transfection of Lv-MIAT shRNA attenuates p65-dependent PUMA expression in the H9C2 cells subjected to H/R

**Figure 2.**
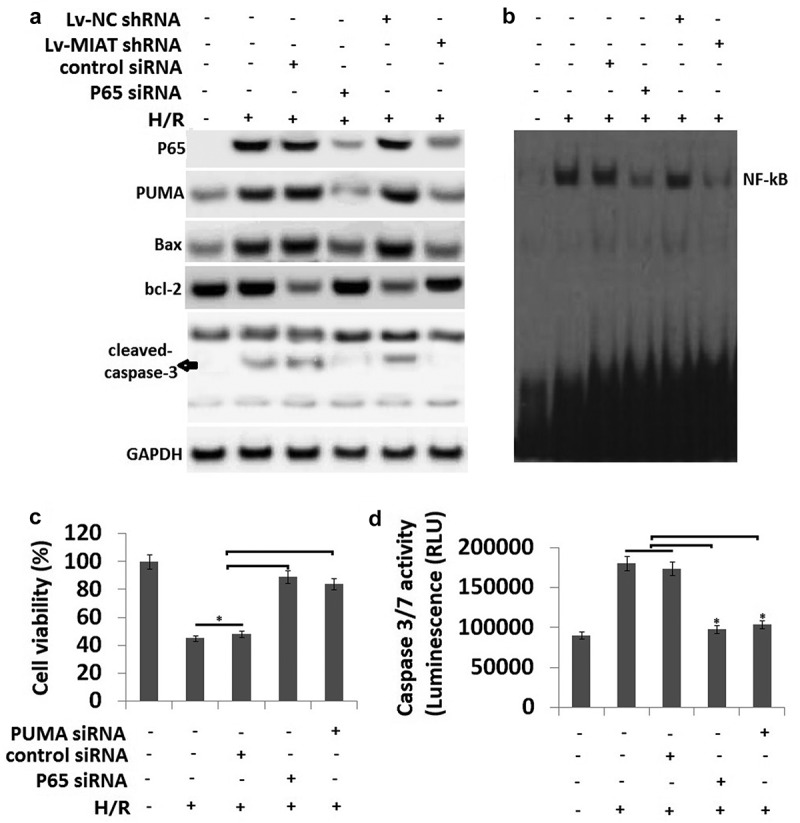
H/R induced p65 nucleus translocation and PUMA signal expression in H9C2 cells. a, Expression of p65, PUMA, bax, bcl-2 and cleaved-caspase-3 were detected by Western blot assay; b, NF-KB activity was detected by EMSA assay; c, Cell viability was detected by MTT; D, Cell apoptosis was detected by caspase-3/7 activity assay. *P < 0.05 compared with indicated groups.

Expression of the NF-κB subunits p65 and NF-κB activity was markedly increased in the H9C2 cells subjected to H/R (2h/24h) compared to the untreated H9C2 cells by western blot assay and EMSA assay (Figure 2(a)-2(b)). In addition, expression of PUMA, bax, cleaved-caspase-3 were upregulated and bcl-2 expression was downregulated in the H9C2 cells subjected to H/R (2h/24h) (Figure 2(a)). Moreover, we found that H/R increased NF-κB activity and P65 expression in the H9C2 cells, which was reversed by p65 siRNA transfection. PUMA, bax, cleaved-caspase-3 expression was also reversed by p65 siRNA transfection, but bcl-2 expression was recovered (Figure 2(a)-2(b)).

To determine whether MIAT are also involved in the regulation of NF-κB and PUMA signals in the H9C2 cells subjected to H/R, H9C2 cells were transfected with **Lv-**MIAT shRNA or **Lv-**NC shRNA and then subjected to H/R (2h/24h). The results showed that MIAT inhibition abolished the effect of H/R on the NF-κB activity (Figure 2(b)) and

P65, PUMA, bax, cleaved-caspase-3 and bcl-2 expression in the H9C2 cells compared with that of the H/R group alone (Figure 2(a)). These data indicate that H/R increased PUMA signals expression via MIAT and NF-κB mediated pathway.

### H/R promotes H9C2 cell injury via P65 and PUMA upregulation

Figure 2(b) shows that H/R markedly decreased cell viability, and targeting p65 or PUMA by siRNA transfection

decreased this effect. Similar changes of caspase-3/7 activity were observed in H9C2 cells used p65 siRNA or PUMA siRNA (Figure 2(d)). Transfection of control siRNA did not affect caspase-3/7 activity in the H9C2 cells subjected to H/R (Figure 2(d)).

### Transfection of Lv-MIAT shRNA reduces infarct size of I/R injured heart

**Figure 3.**
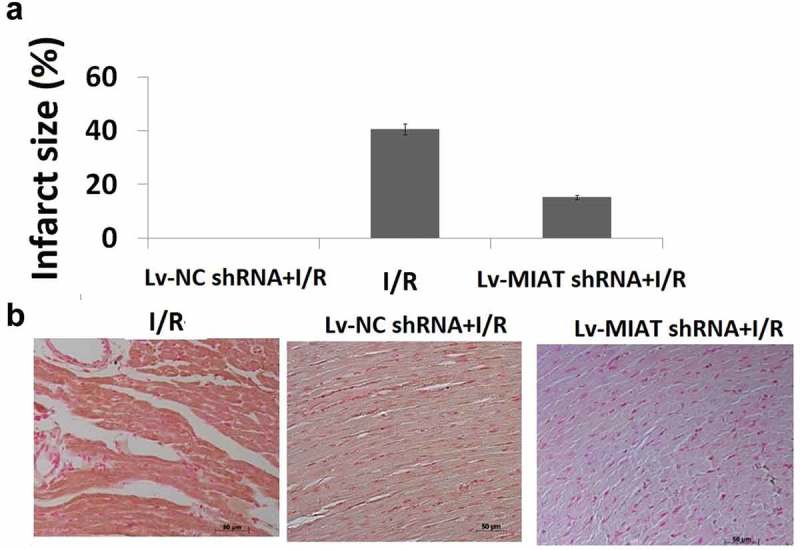
**Targeting MIAT mediated cardioprotective effects in I/R-induced cardiac injury.** a, Representative TTC staining (example slices from one animal in each group) shows infarcted areas in white and non-infarcted areas in red. b, Representative H&E-stained histological images (×200 magnification) of the myocardial sections from different treatment groups, *p < 0.01.

To determine the effects of targeting MIAT on I/R injury, 100 ul of **Lv-**MIAT shRNA or **Lv-**NC shRNA (1 × 10^8^ PFU) was injected through the micro-catheter. **I/R alone** developed a 39.5 ± 4.3% infarct size in the basal portion of ventricle evaluated by TCC staining (Figure 3(a)). However, **transfection of Lv-**MIAT shRNA reduced infarct size to 14.6 ± 1.3% in the **I/R injured heart**, which significantly decreased compared with the **Lv-**NC shRNA groups (Figure 3(a)). Histological examination showed that **I/R injured heart** demonstrated tissue damage, necrosis with massive neutrophil infiltration (Figure 3(b)), and tissue damage was significantly decreased in the **Lv-**MIAT shRNA groups (Figure 3(b)). **Lv-**NC shRNA transfection has not significant affect on the **I/R injured heart** (Figure 3(b)).

### Transfection of lv-MIAT shRNA improves cardiac function in I/R-injured hearts

**Figure 4.**
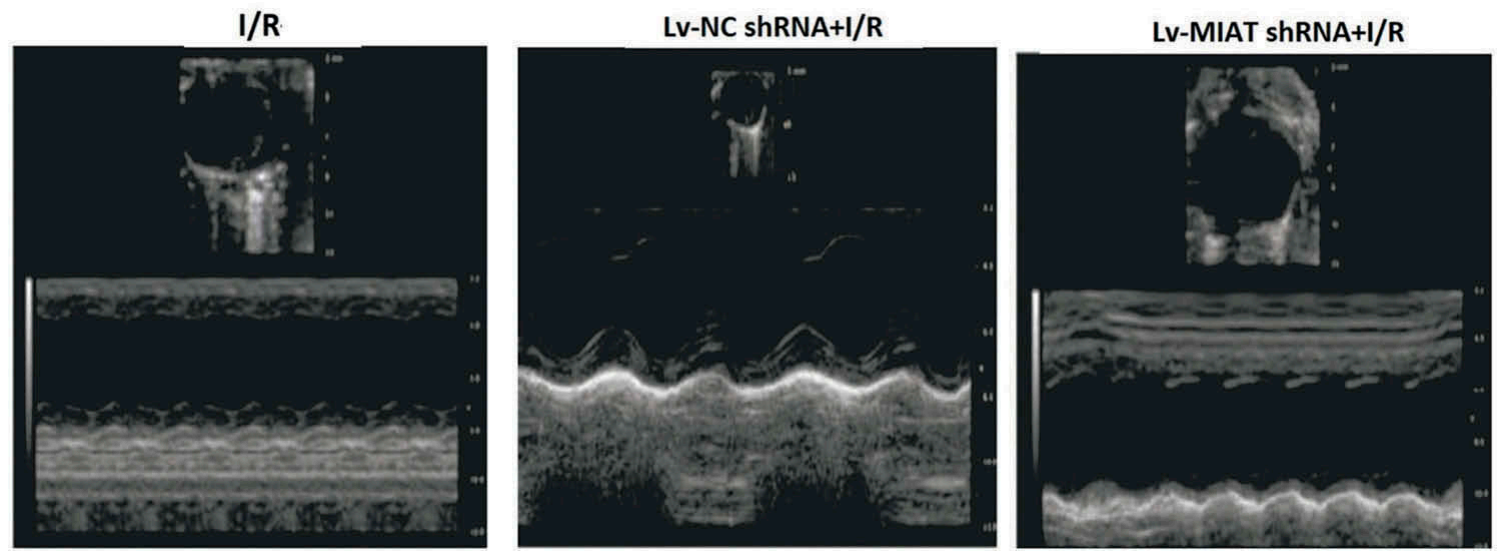
**Effect of targeting MIAT on cardiac functions with echocardiographic evaluation**. Representative echocardiograph recorded from a mouse after I/R in different groups of treatment. vs Lv-NC shRNA, **p < 0.01; vs I/R, *p < 0.05.

Cardiac function was examined by echocardiography after I/R. As shown in Figure 4 and Table 1. The LV fractional shortening (FS), Left ventricular (LV) end-diastolic diameter (LVEDD) and end-systolic diameter (LVESD), and ejection fraction (EF) were significantly increased in the **Lv-MIAT shRNA transfected hearts compared to the**

**Table 1.** The circulating level of **CK-MB** and echocardiography findings in six study groups of animals.

	NC	I/R*	I/R+ shXIST **
CK-MB(ng/ml)	98.3 ± 10.7	260 ± 22.6	176 ± 18.4
**EF (%)**	76 ± 9.3	53.4 ± 7.4	65.3 ± 8.2
**FS (%)**	48 ± 6.3	32.1 ± 6.4	41.4 ± 6.3
LVEDD(cm)	0.56 ± 0.078	0.92 ± 0.054	0.78 ± 0.045
LVESD(cm)	0.34 ± 0.052	0.59 ± 0.038	0.62 ± 0.048

**Lv-NC shRNA transfected hearts**. CK-MB was significantly increased in the **I/R-injured** groups, and **Lv-MIAT shRNA**

significantly decreased the CK-MB levels in the **I/R-injured** groups(Table 1).

### Transfection of Lv-MIAT shRNA suppressed myocardial apoptosis via PUMA

**Figure 5.**
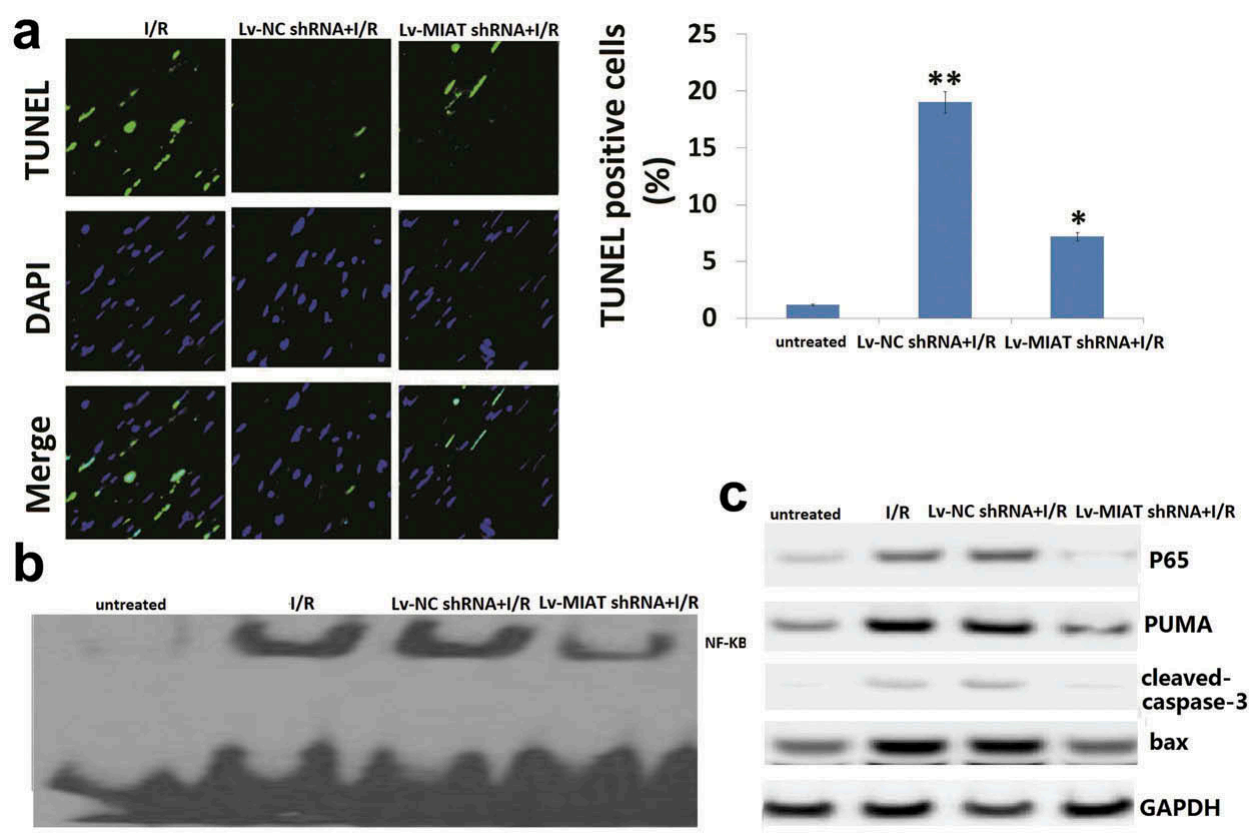
Targeting MIAT protects against I/R-induced cell apoptosis via inhibition of p65 nucleus translocation and PUMA signal expression. a, Representative pictures of sections stained with TUNEL (×400). TUNEL-positive nuclei are shown in green. Blue fluorescence indicated total cardiomyocyte nuclei. Quantitative analysis (percentage of apoptotic cells versus total) is shown in histogram. b, NF-KB activity was detected by EMSA assay; c, Western blots of p65 nucleus translocation, cleaved caspase-3,PUMA and Bax in I/R tissues in the presence or absence of Lv-MIAT shRNA. Vs Lv-NC shNA, **p < 0.01; Vs I/R, *p < 0.05

As shown in Figure 5A, the percentage of TUNEL positive nuclei was significantly increased in the **I/R-injured** hearts compared to the Sham-injured heart (19.5 ± 1.7% vs 0.96 ± 0.2%, *p *< 0.01). However,the number of TUNEL-positive nuclei was significantly decreased by 7.2 ± 0.9% in the **Lv-MIAT shRNA transfected I/R hearts** compared to the **Lv-NC shRNA transfected I/R hearts** (*p *< 0.05). In addition, caspase-3/7 activity was significantly decreased in the **Lv-MIAT shRNA transfected I/R hearts** compared to the **Lv-NC shRNA transfected I/R hearts** (data not shown).

NF-κB activity was decreased (Figure 5(b)), and expression of P65, PUMA, bax, cleaved-caspase-3 was decreased and bcl-2 was increased **in the Lv-MIAT shRNA transfected hearts compared to the Lv-NC shRNA transfected I/R hearts** (Figure 5(c)).

## Discussion

The present study demonstrated that MIAT expression was significantly higher in H9C2 cardiomyocytes in response to H/R stimulation, which was correspondent to severe posthypoxic cardiomyocyte injury. MIAT expression was also significantly higher in mice heart in response to I/R injury, which was correspondent to increased myocardial infarct size and decreased heart function. This suggests that enhancement of MIAT may be a mechanism of myocardial I/R injury. Our findings are in line with several previous studies which showed that targeting MIAT significantly improved cardiac function and reduced myocardial infarction size in different myocardial I/R animal models [20–22]. However, up to now, no study has been conducted to address how inhibition of MIAT couples to multiple signaling molecules and exerts cardioprotective effects.

In the present study, targeting MIAT significantly enhanced cell viability and attenuated H/R-induced LDH release, caspase-3/7 activity, and the expression of cleaved caspase-3 in H9C2 cells. This demonstrated that inhibition of MIAT attenuated H/R-induced cell injury and apoptosis. We demonstrate in vivo in the present study that targeting MIAT attenuates myocardial I/R injury and prevents I/R-induced cardiac dysfunction, and significantly attenuated I/R-induced myocardial apoptosis, indicating that targeting MIAT has a cardioprotective effect against myocardial I/R injury via inhibition of cell apoptosis.

It is well known that constitutive activation of NF‐*κ*B in malignancies may up‐regulate the expression of genes functioning in cell proliferation and anti‐apoptosis, leading to persistent tumour survival [23]. PUMA is the pro-apoptotic gene, by which phosphorylates pro-apoptotic molecule Bax, bad or Bim, leading to dissociation of Bax, bad or Bim from anti‐apoptotic molecule Bcl-2. Wang et al. has recently reported that PUMA is a direct target of NF-κB and mediates TNF-α-induced apoptosis *in vitro* and *in vivo* [24]. Sun et al. has also reported that PUMA was directly activated by p65 through the canonical NF-κB pathway, mediating the apoptotic response to mitotic arrest imposed by aurora kinase inhibition [25]. Dynamic changes in lncRNA expression have been demonstrated upon activation of NF-kB signalling. These changes not only regulate NF-kB activity through direct interaction between lncRNA and NF-kB or its transcripts, but also regulate NF-kB signalling activity indirectly, through upstream components [26–28].

Our data show that targeting MIAT in the H9C2 cells or in the myocardium significantly prevents H/R or I/R-induced H9C2 cells or myocardial NF-κB-binding activity, inhibiting NF-κB nuclear translocation and activation. Importantly, we have observed that the expression of PUMA signals was suppressed by targeting MIAT. PUMA plays a crucial role in the induction of cell apoptosis via activation of bax and caspase-3 expression [12]. We have observed that hypoxia followed by reoxygenation increased the expression of MIAT and increased NF-κB-binding activity and PUMA expression in H9C2 cells. Inhibition of p65 expression prevents H/R-induced increases in PUMA expression. The data indicate that H/R-induced increases in the expression of PUMA might be mediated by NF-κB activation. Targeting MIAT decreased PUMA expression, which is in part mediated by inhibiting NF-κB activation during H/R. Our observation is consistent with previous reports, showing that **NF**-κB is the target for MIAT [29].

Findings of the current study suggest that NFκB is a major player/mediator in H/R or I/R-induced expression of PUMA in H9C2 cardiomyocytes or myocardial. However, besides the NFκB/PUMA signaling, NFκB also controls hundreds of gene expressions which are involved in inflammation, cell growth, differentiation, and cell apoptosis [30]. In particular, accumulating evidences have demonstrated that NFκB promotes cell survival due to the upregulation of anti-apoptotic genes, including A1/Bfl1 [31], Bcl-XL [32], andNr13 [33].Thus, transcriptional factor NFκB exerts both pro- and anti-apoptotic effects in the context of apoptotic stimulus [34]. In the present study, when P65 siRNA was applied to block NFκB activity, PUMA was sequentially blocked, but other downstream signals of NFκB (like NFκB-mediated anti-apoptotic genes)may also be suppressed and this could be the reason why inhibition of PUMA could not completely recover H/R-induced cell injury and apoptosis. We also found targeting MIAT could not completely inhibit H/R or I/R induced cell apoptosis and recover the cell and heart function, potential for off-target effects of MRAT shRNA may exit. The probability for potential off-target effects is likely to be the unique target shRNA we used to target MITA [35]

In concluction, we reveal for the first time that targeting MIAT can directly inhibit cardiomyocyte apoptosis and thus alleviate myocardial reperfusion injury through inhibiting NF-κB activity and PUMA pathway. Therefore, targeting MIAT might represent a new strategy for alleviating myocardial reperfusion injury.
